# AI-Assisted Real-Time Immunoassay Improves Clinical Sensitivity and Specificity

**DOI:** 10.1021/acs.analchem.4c00764

**Published:** 2024-07-09

**Authors:** Diana Lorena Mancera-Zapata, Cynthia Rodríguez-Nava, Fernando Arce, Eden Morales-Narváez

**Affiliations:** † Centro de Investigaciones en Óptica, A. C., Loma del Bosque 115, Lomas del Campestre, León, 37150 Guanajuato, Mexico; ‡ Facultad de Ciencias Químico Biológicas, Universidad Autónoma de Guerrero, Chilpancingo, 39070 Guerrero, Mexico; § Biophotonic Nanosensors Laboratory, Centro de Física Aplicada y Tecnología Avanzada (CFATA), Universidad Nacional Autónoma de México (UNAM), Querétaro 76230, Mexico

## Abstract

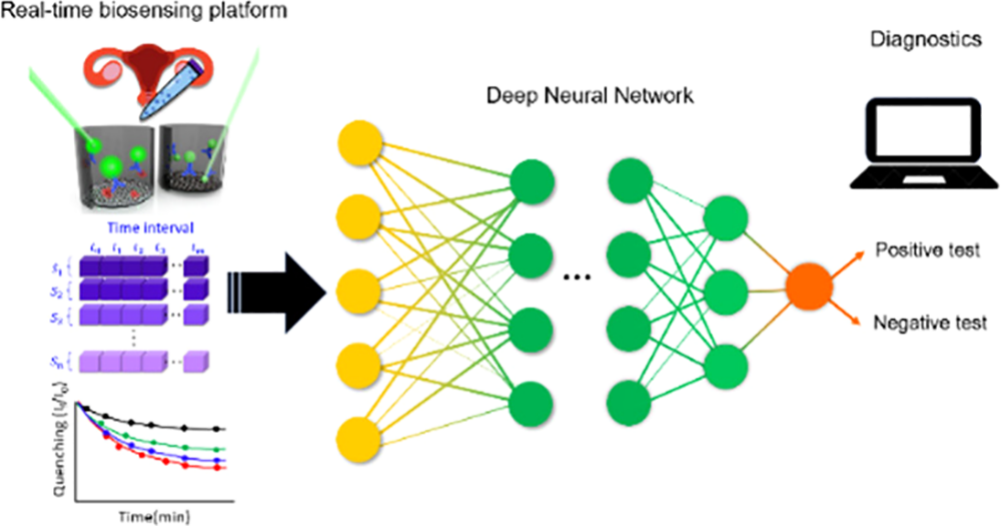

Real-time biosensing systems can interrogate the association between the analyte and the biorecognition element across time. Typically, the resulting data are preprocessed to offer valuable bioanalytical information obtained at a single optimal point of such a real-time response; for instance, a diagnosis of certain medical conditions can be established depending on a biomarker (analyte) concentration measured at an optimal time, that is, a threshold. Exploiting this conventional approach, we previously developed a nanophotonic immunoassay for bacterial vaginosis diagnosis exhibiting a clinical sensitivity and specificity of ca. 96.29% (*n* = 162). Herein, we demonstrate that a real-time biosensing platform assisted by artificial intelligence not only obviates biomarker concentration (i.e., a threshold) determination but also increases sensitivity and specificity in the targeted diagnostic, thereby reaching values of up to 100%.

## Introduction

Biosensors are analytical platforms that can be applied in medical, industrial and agricultural settings, where highly reliable and efficient systems are demanded.^1^ In this context, machine learning algorithms, such as neural networks, are enabling fast processing and analysis of massive data, as well as accurate classification and identification of complex patterns, thus offering new capabilities in many fields, including (bio)sensing and diagnostics.^2−4^ For instance, neural networks have been employed to distinguish patients with leukemia, hepatitis B, or breast cancer from healthy volunteers, respectively.^5^ Furthermore, neural networks can be employed to identify drug-dosed living cancer cells and to discriminate bacterial pathogens.^6−8^

**Figure 1:**
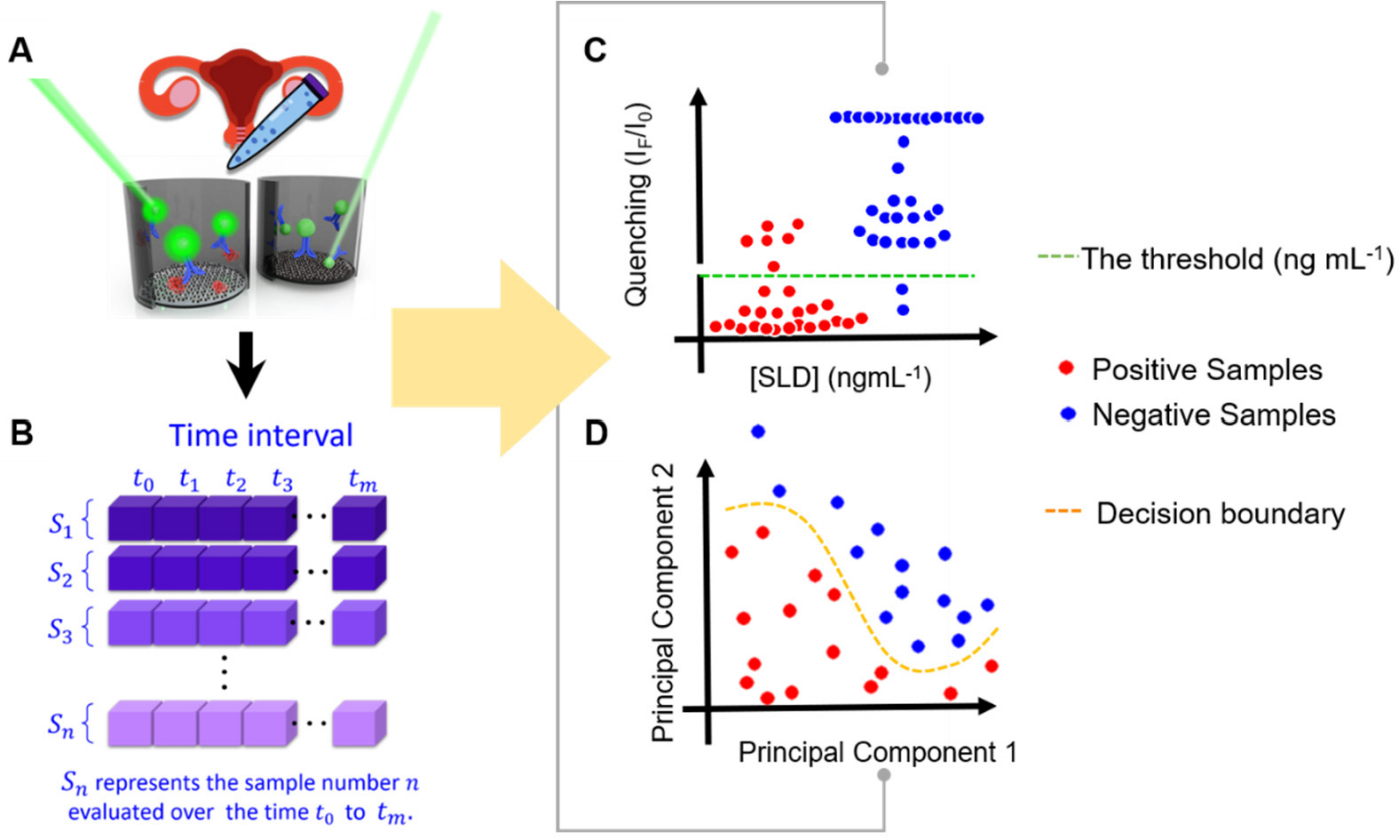
Schematic representation of the AI-assisted bioanalytical platform and comparison with a conventional approach targeting BV diagnosis. **A**. The real-time biosensing platform interrogates the formation of the immunocomplexes. **B**. The real-time biosensing platform yields a data set which is then organized in a matrix with a dimension of *n* × *m*. Row *n* represents the sample of a patient *S*_*n*,_ which is interrogated in real-time using the biosensing platform across time, every 5 min, from *t*_0_ to *t*_*m*_. **C**. Conventional approach targeting BV diagnosis. The red dotted line represents the threshold to determine if BV is positive or negative. **D**. Artificial Intelligence approach. Artificial neural networks are able to generate complex decision boundaries (e.g., orange dotted line), which are then employed to determine BV positive or negative.

Our team has recently developed an advantageous real-time inmunosensing platform based on the fluorescence quenching abilities of graphene oxide-coated microwell plates and fluorescent bioprobes (e.g., fluorophore-tagged antibodies).^9^ These graphene oxide-modified microwells reveal the formation of immunocomplexes since fluorescent bioprobes undergoing immunoreactions have a weak affinity against the graphene oxide-coated surface. Nevertheless, fluorescent bioprobes that are not forming immunocomplexes have a strong affinity against the graphene oxide-modified surface, and the corresponding fluorescence is then quenched, see Figure 1A. Hence, this immunosensing technology can be applied in the detection of biomarkers such as sialidase (SLD), an enzyme overexpressed in vaginal swab samples of women undergoing bacterial vaginosis (BV).^5^ By analyzing the SLD levels in 162 clinical samples and establishing a threshold around 25 ng mL^–1^ to determine BV negative or BV positive, our real-time immunosensing platform was proven to be useful for BV diagnosis, achieving a clinical sensitivity/specificity of approximately 96%. Accurate BV diagnosis is a clinically relevant issue since BV is a common infection in reproductive-age women and may lead to dramatic consequences, including pelvic inflammatory disease, preterm premature rupture, low weight in the newborn, miscarriage, and amniotic fluid infection that can persist (even after birth) and lead to the death of the newborn.^5^ BV is mainly diagnosed by clinical observations (via Amsel’s criteria and or the Nugent scoring system) executed by a healthcare specialist, which are time-consuming but display high clinical specificity and sensitivity. In addition, the market offers colorimetric methods for VB diagnosis.^10^ However, given their qualitative response, the clinical specificity and sensitivity of these methods may depend on the observations of the users (Table S1).

Real-time biosensing systems generate a series of data related to the association between the analyte and the biorecognition element. Typically, the resulting data are processed to get analytical information, such as analyte concentration, obtained at an optimal single point of such a real-time response.^11^ Herein, we demonstrate that this series of data can be useful to feed artificial neural networks and perform accurate BV diagnosis; see Figure 1. Different artificial neural network architectures were studied to optimize a binary classification task (BV positive or negative) and eventually enhance clinical sensitivity and specificity. The employed neural networks include dense neural networks (DNNs),^12^ convolutional neural networks (CNNs),^13^ and long short-term memories (LSTMs)^14^ since they have been reported to exhibit excellent performance in classification tasks.

## Experimental Section

### Nanophotonic Immunoassay for Sialidase Detection

**Figure 2:**
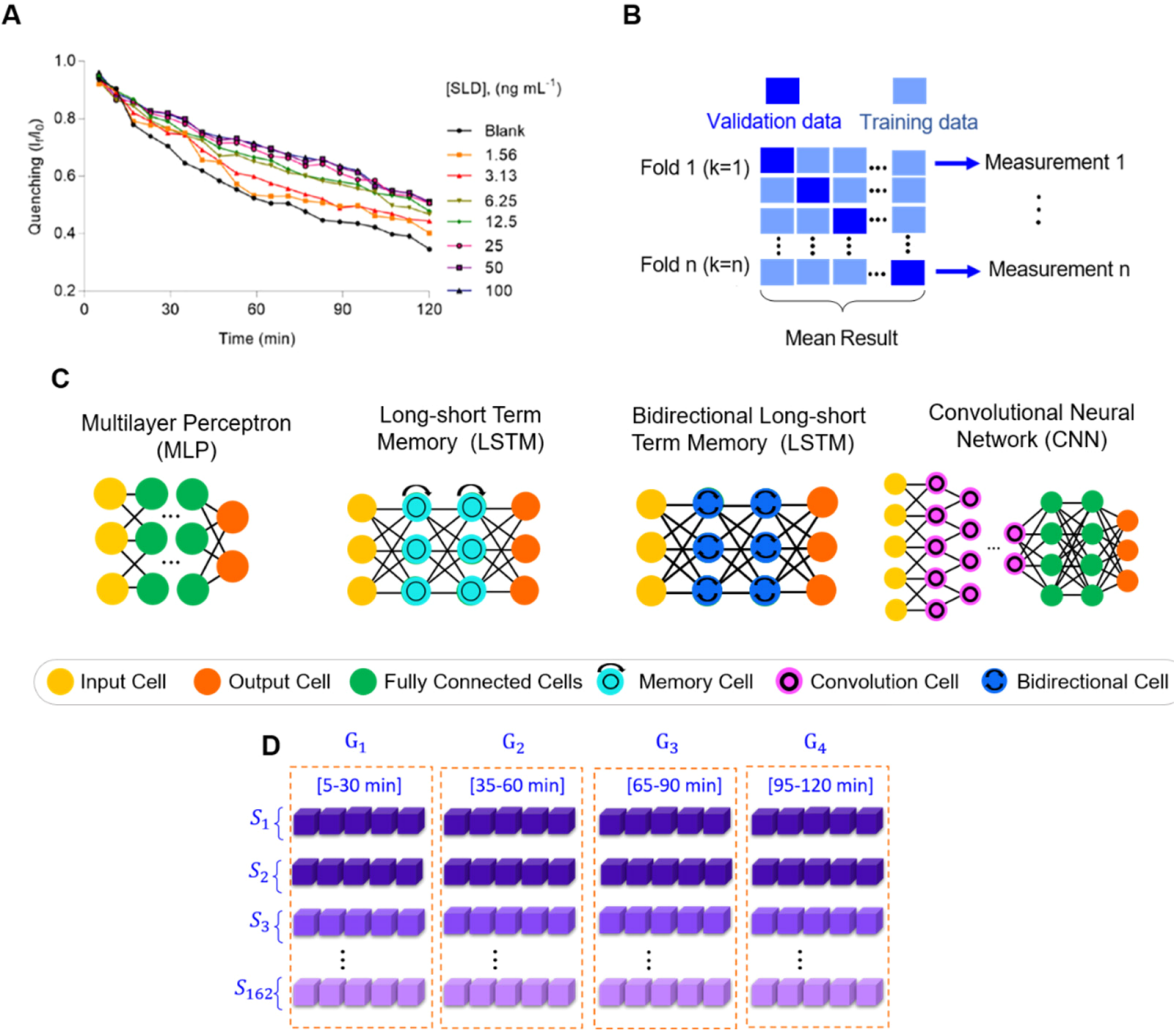
**A**. Type of data and tools utilized in this research. **A**. Kinetic analysis of fluorescence quenching depends on the SLD concentration. Figure adapted with permission from ref (5). Copyright © 2021 American Chemical Society. **B**. K-fold cross validation. **C**. Neural Network architectures. **D**. Data set splitting.

According to the previously described biosensing mechanism, the nanophotonic immunoassay reveals immunocomplex formation and analyte concentrations according to specific fluorescence quenching values. As previously reported,^5^ 162 clinical samples were classified as BV positive (BV+) or BV negative (BV-) according to the Amsel criteria, a standard procedure to diagnose BV (54 samples were BV+ and 108 were BV-). The clinical samples were mixed with the photoluminescent probes in a GO-coated microwell to perform a kinetic analysis of fluorescence quenching levels. Figure 2A depicts an example of this kinetic analysis.

### Data Set Arrangement

Showing intra-assay coefficients of variation (CVs) ranging from 0.14% to 3.53% and interassay CVs from 5.82% to 7.94%, the precision of the nanophotonic immunoassay was proven acceptable for immunosensing.^5^ The formation of the data series depended on the fluorescence quenching ratios obtained by means of the nanophotonic immunoassay: *I*_*f*_/*I*_0_. Here, *I*_0_ represents the fluorescence intensity of the analyzed sample at time 0, while *I*_*f*_ represents the fluorescence intensity of the same sample at time *f*. The data collection involved 5 min intervals over a period of 120 min. We previously demonstrated that this interval represents a suitable condition to study real-time immunosensing systems, since with these data we are able to model protein kinetics accurately and determine protein kinetics constants.^11^ Therefore, the series of data was arranged in a matrix of 162 rows and 20 columns, where 162 is the number of samples and 20 corresponds to the resulting measurements (features) of the respective *I*_*f*_/*I*_0_ values, see Figure 1B. This series of data is publicly available on the GitHub repository.^15^

In order to analyze the temporal patterns within the collected data and find optimal time intervals for the binary classification process (BV+ or BV-), we partitioned the data set into four groups, denoted as G_1_, G_2_, G_3_, and G_4_. Each group encompasses five measurements, representing intervals of 25 min. Consequently, the first group corresponds to the initial 25 min, the second covers the period from 25 to 50 min, and so forth, until the entire experimental duration is accounted for (i.e., 120 min). Importantly, this data arrangement offered an even distribution of the data within the explored groups.

### 2D Visualization of the Data

Before training the neural network architectures, we employed principal component analysis (PCA) to diminish data dimensionality and generate a 2D data distribution plot; this allowed for the observation of data correlation according to the analytical behavior. A script for PCA as well as fluorescence level data was shared on GitHub repository.^15^ See Section 1 in the Supporting Information to find a detailed explanation of PCA. Additionally, a classifier known as the “Nearest Centroid Classifier” was used to determine the separation between classes quantitatively.

### Training Process

We employed the K-fold cross-validation technique, which is an iterative process that consists of dividing the data set into K folds or subsets, using K-1 of them for model training and the remaining one for validation in each iteration. This approach allows for a robust and generalized evaluation of the model by considering different combinations of training and validation of data at each step of the process. A visual representation of this technique is provided in Figure 2B.

Due to the amount of our data, the K-fold cross-validation technique was most effective when using k = 10, i.e. during the training process the data were divided into 10 folds, so that, having 54 positive and 108 negative samples, each of the partitions had 10 negative and 5 positive samples to be evaluated. This information was crucial for interpreting the confusion matrices later on.

Prior to the training of the neural architectures, the data set was normalized according to eq 1, as this contributes to the convergence of the networks and thus to the significant improvement of the results.
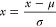
1Here *x* is the input data, μ is the mean, and σ is the standard deviation of the fluorescence quenching levels of a given sample.

The proposed neural architectures, MLPs, LSTMs, and CNNs, were trained as a supervised learning task,^16^ i.e., each sample corresponds to a label (BV+ or BV-) according to the aforementioned Amsel criteria.^5^ In the script developed for this work, positive samples (BV+) were represented by a vector of ones (54 × 1) and negative samples (BV-) by a vector of zeros (108 × 1). Both vectors were joined to form a new vector with dimensions of 162 × 1. In this classification process, no outliers were found or discarded.

Neural networks are composed of organized layers of neurons or cells, each with a specific functions. These layers, including the output layer and hidden layers (HL), process input information such as fluorescence quenching level measurements to categorize each sample as BV+ or BV-. The goal is to establish a relationship between each sample and its respective class. Figure 2C illustrates the various neural architectures used along with their corresponding layers. A brief description of the operating principle of these neural networks can be found in Section 2 of the Supporting Information.

The performance of the models was optimized by changing different hyperparameters, such as the number of layers, number of neurons, activation functions, and regularization rate, among others. Table S2, see the Supporting Information, shows the performance of different architectures by changing their different hyperparameters. Twelve MLPs configurations were explored; for this specific architecture, it was necessary to use “dropout” as a regularization technique since in some cases overfitting occurred.^17^ The performance of four LSTMs and four bidirectional LSTMs (B-LSTMs) was also explored, since these types of neural architectures are widely used with temporal sequence data,^18^ similar to our data set.^19^ Finally, as convolutional architectures have been shown to be suitable for addressing sequential prediction tasks.^20−22^ we employed four 1D convolutional networks (1D-CNN). Table 1 summarizes the optimal parameters such as the number of layers, number of neurons, etc., for those models that demonstrated superior performance, respectively.

**Table 1:** Summary of the Performance of the Proposed Architectures, Assessed with 10-Fold Cross-Validation^a^

Parameter	MLP	LSTM	BLSTM	1D-CNN
HL 1 (*N*° of neurons)	FCL:30	LSTMs:20	BLSTM:30	**CNN:20**
Dropout	0.2	0	0	**0**
HL 2 (*N*° of neurons)	FCL:30	LSTMs:20	BLSTM:30	**CNN:20**
Dropout	0.2	0	0	**0**
HL 3 (*N*° of neurons)	0	FCL:20	FCL:20	**FCL:20**
Accuracy in training (%)	98	100	100	**100**
Accuracy in validation (%)	94	98.0	98.0	**100**
Sensitivity (%)	99.6	99.6	99.5	**100**
Specificity (%)	99.9	99.9	99.9	**100**

a The abbreviation HL refers to the hidden layer, and the characters in bold represent the best performance.

To assess the effectiveness of the models, we considered validation and training accuracy percentages, as well as sensitivity and specificity percentages; see Table 3 and Tables S3–S8 in the Supporting Information. Evaluation metrics such as Confusion Matrix and F_1_ score were also used and are described in detail in Section 3 of the Supporting Information. The respective confusion matrices and F_1_ scores can be found in Figures S3–S7 and Tables S3–S6 in the Supporting Information.

### Feature Selection

Once an optimal architecture for the BV classification task was identified, we used 1D-CNN to highlight the most relevant features of the data and determine the optimal time interval. To this end, we employed the data groups previously used in PCA, G_1_, G_2_, G_3_, and G_4_, see Figure 2D.

To optimize the employment of the data set and gain insights into the behavior of the groups, we deployed the 1D-CNN model and systematically combined the four individual data sets. For instance, G_1,2_ denotes the merger of groups G_1_ and G_2_, G_2,3_ denotes the merger of G_2_ and G_3_, and so on.

### Determination of Clinical Sensitivity and Specificity

The sensitivity, specificity, precision, and accuracy values of our model were derived from the number of false positives, false negatives, true positives, and true negatives obtained during the classification process. In our script,^15^ we used the Python library *sklearn.metrics* which employs the mathematical expressions provided in eqs 1S-4S, see the Supporting Information. In addition, we calculated the F_1_ score (an evaluation metric), which combines the precision and sensitivity (also known as recall) scores, following eq 5S, see the Supporting Information.

## Results and Discussion

As previously discussed, using the Amsel criteria as a reference for BV determination, BV was diagnosed with clinical specificity and sensitivity of ca. 96%, respectively, by means of a conventional analysis of the data offered by our real-time immunoassay.^5^ Following the methods detailed in the Experimental Section, we demonstrate that the series of data delivered by a real-time immunoassay can be useful to feed artificial neural networks and outperform the aforementioned specificity and sensitivity.

**Figure 3:**
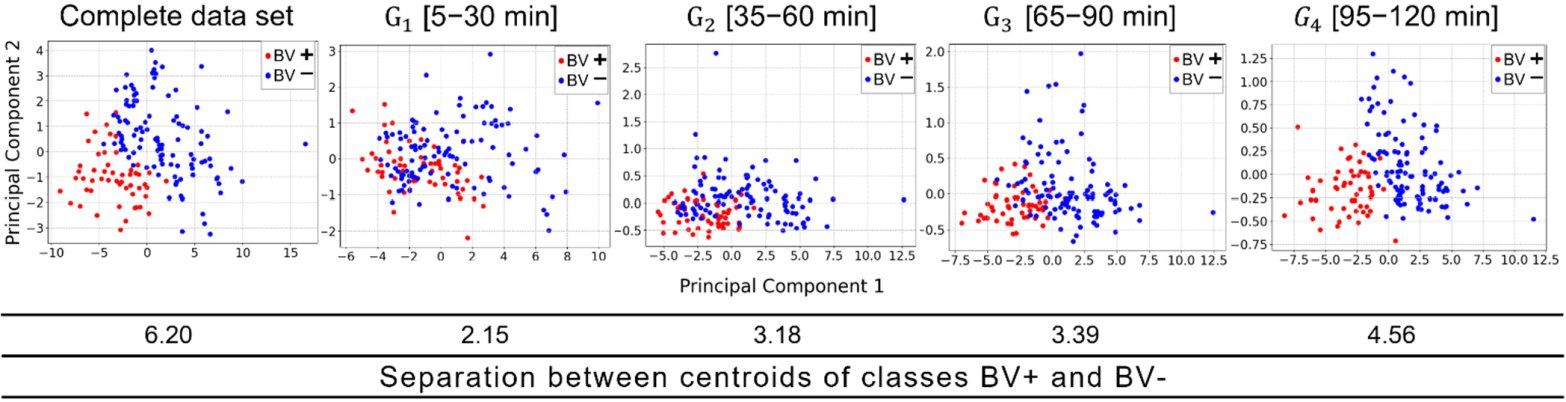
PCA and distances between the classes BV+ and BV- (complete data set and different times data sets).

First, PCA allowed for the visualization of the 2D distribution of our data set as a function of the principal components over the execution of the nanophotonic immunoassay. Figure 3 illustrates how the samples correlate with each other throughout time. In particular, we observed that the G_4_ group composed by the last time interval (95–120 min) displays a clear division between the BV+ and BV- samples. The usage of the nearest centroid classifier allowed us to quantitatively confirm that, over time, the data set shows a progressing difference between the BV+ and BV- classes. In fact, the separation between the centroids of each class increased over time, reaching a maximum separation at 120 min (see Figure 3).

Table S2 (see the Supporting Information) displays the performance of the 24 neural networks that were evaluated in this research according to the Experimental Section. Table 1 summarizes the best performance of the different neural networks trained in this work. As shown in Table 1, the 1D-CNN model, which was comprised of two 1D-CNN layers with 20 neurons each in the first and second hidden layers and a third layer with 20 fully connected cells, see Figure S2 in the Supporting Information, was identified as the most effective model for BV diagnosis.

Consequently, the sensitivity and specificity of BV diagnosis accounted for 100% using this 1D-CNN configuration, as observed in the last column in Table 1.

Furthermore, as depicted in Table 2, LSTM and 1D-CNN architectures exhibited the highest accuracy to perform the BV diagnosis. The mean F_1_ score^23^ for LSTM architecture was 0.97, whereas the mean F_1_ score for CNN architecture was 1.0. Additionally, the B-LSTM architecture obtained a 0.96 F_1_ Score, while the MLP architecture obtained 0.92. These results are consistent with a previous work that compared the performance of CNNs, MLPs, and LSTMs as time series forecasting methods, where CNNs and LSTMs were shown to yield better classification tasks than MPLs.^24−27^

**Table 2:** F_1_ Score for Each Fold in the MLP, LSTM, B-LSTM, and 1D-CNN Architectures

	F_1_ score			
Fold	MLP	LSTM	B-LSTM	1D-CNN
1	1.00	**1.00**	1.00	**1.00**
2	1.00	**1.00**	1.00	**1.00**
3	0.91	**1.00**	0.91	**1.00**
4	0.89	**0.89**	0.89	**1.00**
5	0.83	**0.91**	0.91	**1.00**
6	0.91	**1.00**	1.00	**1.00**
7	0.91	**0.89**	1.00	**1.00**
8	0.80	**1.00**	1.00	**1.00**
9	1.00	**1.00**	0.91	**1.00**
10	0.91	**1.00**	1.0	**1.00**
**Mean**	**0.92**	**0.97**	**0.96**	**1.00**

Confusion matrices in Figures S3–S6, aside from being an evaluation metric, provided a graphical representation of the model performance in a classification task; i.e., Figure S4 shows that the 1D-CNN architecture achieves 100% accuracy in the classification task since it did not obtain false positives or false negatives in any of the 10 folds.

As discussed above, PCA demonstrated that group G_4_ offered a less overlapping area between BV+ and BV- samples. This is reflected in a clear boundary between BV+ and BV- groups. This finding is consistent with the validation and training percentages (see content highlighted in bold in Table 3), as well as with the conclusions drawn in the conventional method for BV diagnosis based on a concentration threshold, where the biosensing platform showed superior performance, that is, only considering those data collected at the end of the immunoassay (120 min).

**Table 3:** Performance of the Best Architecture, the 1D-CNN, for the Time Groups G_1_, G_2_, G_3_, and G_4_^a^

Group	Accuracy in training (%)	Accuracy in validation (%)	Sensitivity in all data (%)	Specificity in all data (%)
*G*_1_	81.7	70.0	76.0	82.5
*G*_2_	84.7	77.3	76.8	87.9
*G*_3_	90.8	88.0	83.5	94.5
***G***_**4**_	**98.7**	**97.3**	**99.6**	**98.0**
*G*_1,2,3,4_	100	**100**	100	100

a The most relevant data are marked in bold.

**Figure 4:**
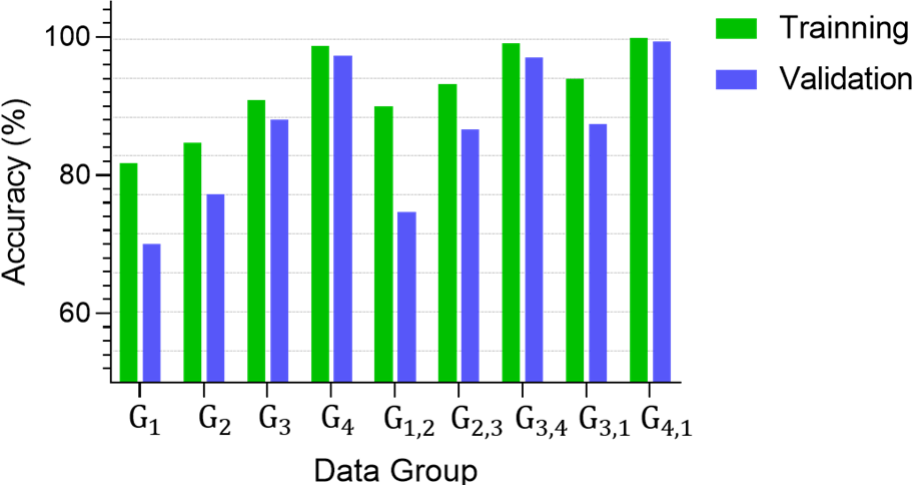
Bar chart depicting the performance of the 1D-CNN architecture fed with different combinations of data groups.

We also explored the performance of our AI-assisted approach by using combinations between groups, which showed that group G_4,1_ offered the highest percentages in training and validation accuracy, when compared with the other group combinations, see Table S7 in the Supporting Information. Actually, the performance with G_4,1_ was better than that obtained using the G_4_ group; particularly, in terms of training and validation accuracy, G_4_ obtained values of 98.7% and 97.3%, respectively, while G_4,1_ obtained 99.8% and 99.3%, respectively, suggesting that the combination of initial and final measurements provided the most relevant information in this AI-assisted approach. A visual representation of this experiment can be found in Figure 4.

Importantly, in order to further evaluate the performance of the 1D-CNN model, particularly with unseen data, another experiment involving a test set comprising unseen data was executed. Hence, we evaluated the model by allocating 15% of our data set for validation, another 15% for testing, and the remaining data set for training (the data were randomly assigned, respectively), where the corresponding F1 score was 1.00 and the sensitivity as well as the specificity accounted for 100%, thereby confirming the advantageous performance of the model; see Table S8 and Figure S7 in the Supporting Information.

It is worth discussing that the dependence on the number of analyses is a crucial factor in the field of AI-assisted biosensors. In this context, the optimal size of the training set will be determined by the nature of the addressed problem and the characteristics of the data, such as dimensionality and quality (ex. signal-to-noise ratio). Currently there are other strategies such as data augmentation or transfer learning which is a relevant resource for deep learning applications with limited data scenarios.^28^ Our approach was limited by the availability of clinical samples; however, we implemented strategies to mitigate these constraint, such as cross-validation, regularization (dropout) techniques, and hyperparameter tuning.

## Conclusions

We demonstrated that clinical sensitivity and specificity of a real-time biosensing system can be improved by implementing neural networks, especially highlighting the performance of CNN and LSTM architectures in classifying time-varying data sets compared to MLPs, and showing their ability to understand and remember time-series features. The resulting AI-assisted approach not only eliminated the need to determine biomarker concentration (or a threshold) to reach a diagnosis through a real-time immunoassay but also significantly improved clinical sensitivity and specificity, reaching values of up to 100% in a binary classification. We also demonstrated that machine learning approaches are also useful to spot optimal test times of a real-time biosensing platform. Although the sensitivity/specificity increased from 96% to 100%, we consider this improvement to be relevant, especially when applied to a large population, which could mean an accurate diagnosis of hundreds (or more) of patients. All in all, by taking advantage of a biosensing generating real-time data on the binding interaction of the biorecognition element and the analyte (biomarker), the proposed AI-assisted approach can be adapted to diagnose other medical conditions in a smart and accurate fashion.

## Supporting Information Available

Description of the employed AI tools and data supporting the reported results are detailed (PDF)
