# Supplementary material for: AI-Assisted Real-Time Immunoassay Improves Clinical Sensitivity and Specificity
Source: Anal Chem. 2024 Jul 9;96(34):13756–61. doi: 10.1021/acs.analchem.4c00764 (PMC11359380; doi:10.1021/acs.analchem.4c00764)
Supplement: Supplementary file 1 — ac4c00764_si_001.pdf [file ac4c00764_si_001.pdf]

Supporting Information

AI-Assisted Real-Time Immunoassay Improves Clinical Sensitivity and Specificity

Diana Lorena Mancera-Zapata[1], Cynthia Rodríguez-Nava[1,2], Fernando Arce[1]*, Eden Morales-Narváez[3]*

[1] Centro de Investigaciones en Óptica, A. C., Loma del Bosque 115, Lomas del Campestre, León, 37150, Guanajuato, Mexico.

[2] Facultad de Ciencias Químico Biológicas, Universidad Autónoma de Guerrero, Chilpancingo 39070, Guerrero, Mexico.

[3] Biophotonic Nanosensors Laboratory, Centro de Física Aplicada y Tecnología Avanzada (CFATA), Universidad Nacional Autónoma de México (UNAM), Querétaro, 76230, Mexico

* farce@cio.mx, eden@fata.unam.mx

Table of contents

Section 1. Principal Component Analysis (PCA).

Section 2. Neural Network Architectures employed.

Section 3. Evaluation metrics**.** Confusion Matrix. F_1 score.

Figure S1. Confusion matrix

Figure S2. Neural architecture for Bacterial Vaginosis classification.

Figure S3. Confusion matrices for each fold in MLP architecture.

Figure S4. Confusion matrices for each fold in CNN architecture.

Figure S5. Confusion matrices for each fold in LSTM architecture.

Figure S6. Confusion matrices for each fold in B-LSTM architecture.

Figure S7. Confusion matrix including unseen data (test set).

Table S1. Sensitivity and specificity of well-established bacterial vaginosis diagnostic methods.

Table S2. Performance of the evaluated architectures (assessed with 10-fold cross-validation).

Table S3. F_1 Score for each fold in MLP architecture.

Table S4. F_1 Score for each fold in CNN architecture.

Table S5. F_1 Score for each fold in LSTM architecture.

Table S6. F_1 Score for each fold in B-LSTM architecture used.

Table S7. Performance of the 1D-CNN architecture fed with different combinations of data groups.

Table S8. Results of the training process using unseen data.

Section 1.

Principal Component Analysis (PCA)

Simplified datasets are easy to study and visualize, which are also recommended to feed machine learning algorithms and analyze data quickly and efficiently. PCA is a dimensionality reduction method, which is frequently used to simplify data sets by reducing the number of features (components) of large datasets. A combination of the initial variables is then used to create each dimension or principal component that results from PCA.[1] PCA facilitated the 2D visualization of the data recorded via the real-time immunoassay.

Section 2.

Neural Network Architectures employed

Dense neural networks (DNNs), also known as multilayer perceptrons (MLPs) or feedforward neural networks, typically consist of three layers: the input, hidden, and output layers. These layers are also called fully-connected layers (FCLs), where each neuron is connected to every neuron in the preceding layers and the layers are stacked together. In DNNs architectures, information flows from the back to the front, layer by layer. Within each layer, the network computes a weighted sum of the previous inputs and weights, followed by the application of a non-linear function. This process continues until reaching the output layer, which provides the network's prediction. DNNs are commonly employed for classification and regression tasks.[2]

Convolutional neural networks (CNNs) are composed of convolutional, pooling, and dense layers, with alternating convolutional and pooling layers. In a CNN, convolutional layers perform cross-correlation operations and work as feature extractors. The pooling layers conduct a down-sampling or dimensional reduction of the network features, being max-pooling the most common pooling layers. This layer promotes the loss of information and reduces the risk of overfitting, increases efficiency, and decreases complexity. The output is a dense layer or fully connected layers that

yields classification and regression of patterns, which are based on the features extracted by the previous layers. [3]

Long short-term memories (LSTMs) are a recurrent neural network architecture designed to handle long-term sequential data and dependencies. LSTM improve the limitations of conventional RNNs by adding memory cells, which are able to retaining information over extended periods. These architectures comprise memory cells, input gates, output gates, and forget gates. The memory cells store and update information, though the gates regulate the flow of information within the network. The input gate controls the information entering the memory cells. The forget gates determine which information should be discarded. The output gates decide which information go to the "memory cells". A distinguishing feature of LSTM is their ability to selectively retain or forget information based on the context and relevance of the input sequence. This enables LSTM to effectively capture long-term dependencies in sequential data, making them well-suited for speech recognition, time series analysis, and natural language processing.[4]

Section 3 . Evaluation metrics

These metrics are evaluation measures used to determine or evaluate the performance of a classification model.

Confusion Matrix

Confusion matrices are commonly used in binary and multi-class classification problems. They allow us to visually and quantitatively evaluate the performance of a model. [5]

An example of a binary classification confusion matrix is depicted in the Figure below.

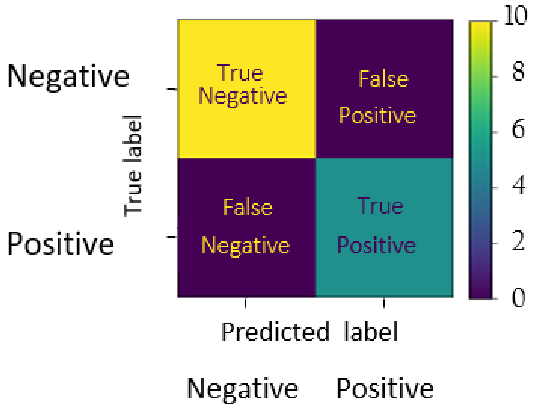

Figure S1. Confusion Matrix for binary classification. The term, True Positive (TP) refers to a sample belonging to the positive class being classified correctly, whereas, True Negative (TN) refers to a sample belonging to the negative class being classified correctly. Additionally, False Positive (FP) refers to a sample belonging to the negative class but being classified wrongly as a positive sample, and False Negative (FN) refers to a sample belonging to the positive class but being classified incorrectly as belonging

to the negative class. It is important to note that the horizontal axis represents all prediction and the vertical axis represents all true values.

Using the number of false positives and false negatives, we can compute the sensitivity, specificity, precision, and accuracy of our model based on the mathematical expressions: [6]

$$\%\,\text{Recall (Sensitivity)} = \frac{TP}{TP + FN} \times 100 \qquad \text{(Eq. 1S)}$$

$$\%\,\text{Specificity} = \frac{TN}{TN + FP} \times 100 \qquad \text{(Eq. 2S)}$$

$$\%\text{Precision} = \frac{TP}{TP + FP} \times 100 \qquad \text{(Eq. 3S)}$$

$$\%\,\text{Accuracy} = \frac{TP + TN}{Total\ Sample} \times 100 \qquad \text{(Eq. 4S)}$$

Note that accuracy can be calculated by taking average of the values lying across the main diagonal.

In order determine the performance of the neural networks, the corresponding confusion matrixes were generated by means of the sklearn.metrics Python library.[6] (Figure S5-S8).

F_1 score

Precision and sensitivity are two crucial metrics, but they are often in conflict. Precision focuses on the accuracy of positive predictions, while sensitivity focuses on the ability of the model to capture all positive cases. The F_1 score provides a balance between these two metrics.

Performance in classification task of our model were also calculated (Table S2-S5). This evaluation metric combines the precision and sensitivity (also called recall) scores of a model according to the expression below which is an harmonic mean: [7]

$$F_1\ score = \frac{2 \times Precision\ \times Recall}{Precision + Recall} \qquad \text{(Eq. 5S)}$$

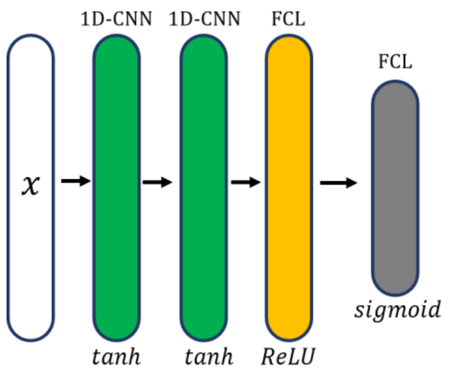

Figure S2. Neural architecture for Bacterial Vaginosis diagnosis. The 1D-convolutional architecture is composed of three hidden layers (HLs) and one output layer. The first two HLs are 1D CLs and employs hyperbolic tangent activation functions with 30 kernels of size 3. The third hidden layer is a FCL composed of 20 neurons that employs ReLU functions. The output layer is made of one single sigmoid activation function, because we are dealing with a binary classification problem.

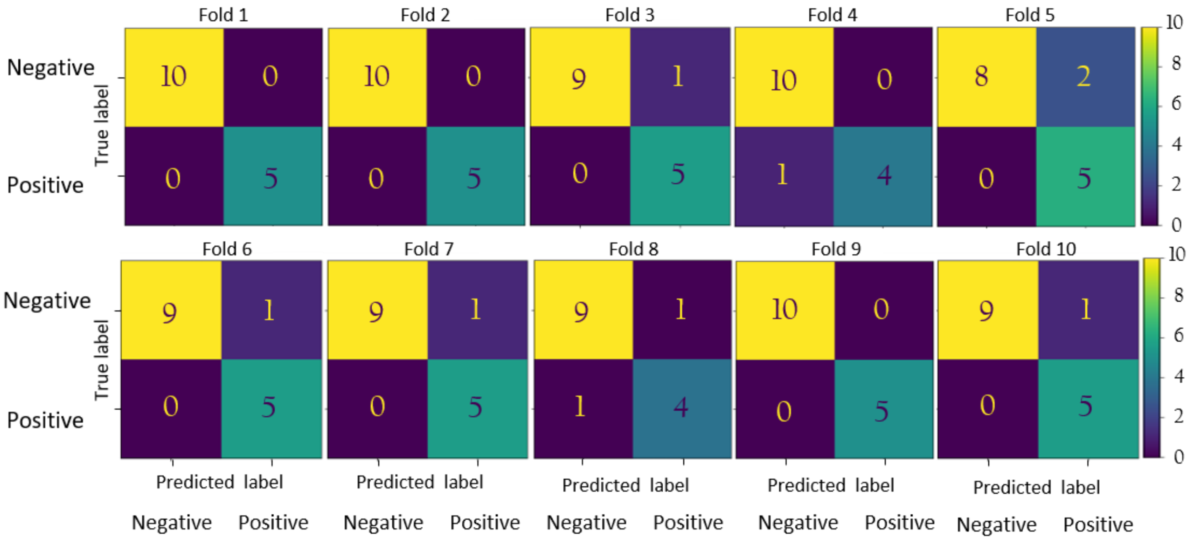

Figure S3. Confusion matrices for each fold in MLP architecture. MLP architecture made a classification without errors in folds 1 and 2, whereas in folds 3, 6, 7 and 10, it classified a negative sample as positive, in fold 4 it classified a positive sample as negative, in fold 5 it presented two false positives and finally, in fold 8 it presented a false positive and a false negative.

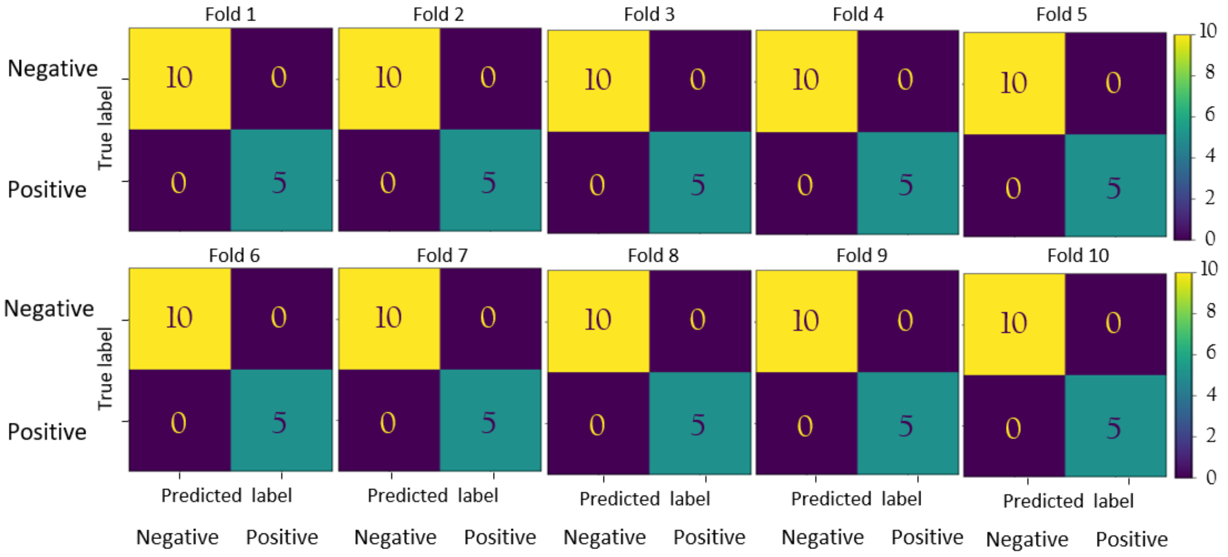

Figure S4. Confusion matrices for each fold in CNN architecture. 1D-CNN architecture achieves 100% accuracy in the classification task since it did not obtain false positives or false negatives in any of the 10 folds.

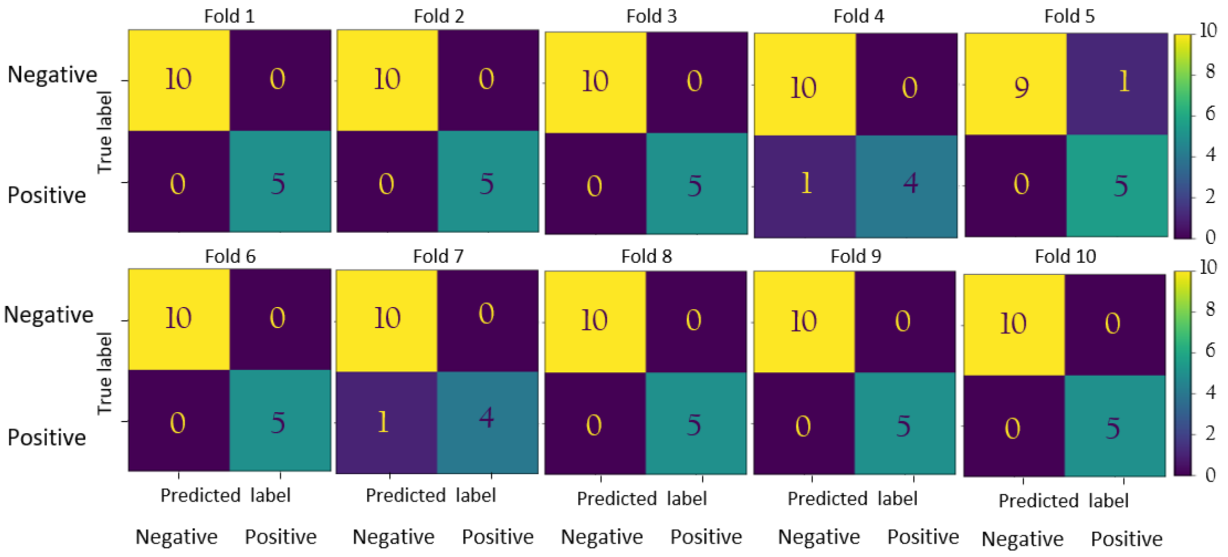

Figure S5. Confusion matrices for each fold in LSTM architecture. LSTM architecture in folds 1, 2, 3, 6, 8, 9 and 10 correctly classifies both the 10 negative samples and the 5 positive samples. On the other hand, in fold 4, out of 5 positive samples, it classifies 1 as negative and in fold 5 out of 10 negative it classifies 1 as positive.

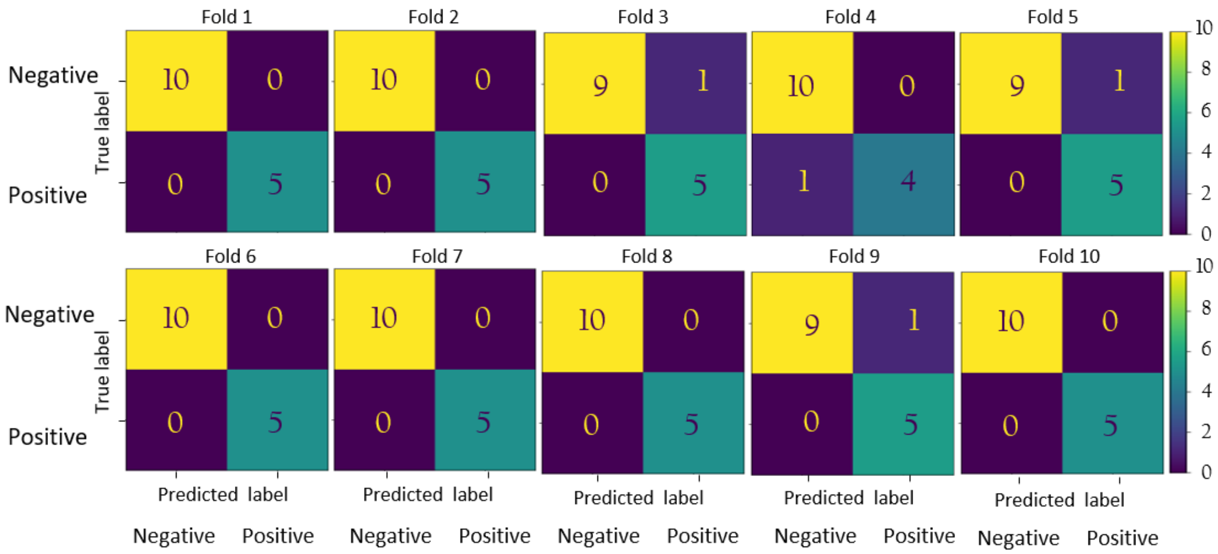

Figure S6. Confusion matrices for each fold in B-LSTM architecture.

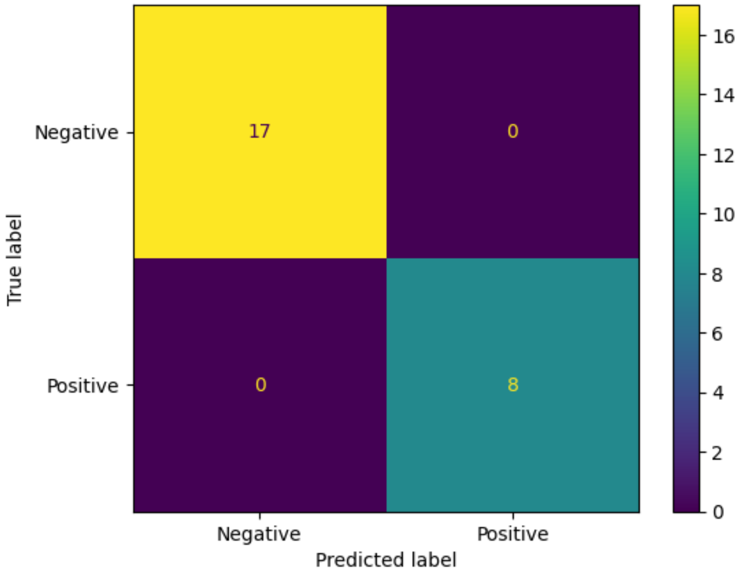

Figure S7. Confusion matrix including unseen data (test set). 70% of the data was used for the training set, 15% for the validation set, and 15% for the testing set. Employed model: 1D-CNN.

Table S1. Sensitivity and specificity of commercial bacterial vaginosis diagnostic methods.[9]

Method	Sensitivity (%)	Specificity (%)
Microscopy		
Amsel's criteria	37 -70	94-99
Nugent scoring system	89-95	83 -94
Molecular		
NuSwab®<br>SureSwab BV DNA<br>BD Max	90.5 -96.7	85.8 - 95
Metabolome/Protemoe		
FemExam®	91	62
OSOM®	96	98

Table S2. Performances of the tested architectures assessed with 10-fold cross-validation.

**N°**	**Hidden layer 1 Number of neurons**	**Dropout**	**Hidden layer 2 Number of neurons**	**Dropout**	**Hidden layer 3 Number of neurons**	**Dropout**	**Accuracy in training (%)**	**Accuracy in validation (%)**	**Sensitivity in all data (%)**	**Specificity in all data (%)**
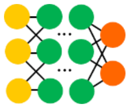	**MLP (Multilayer Perceptron)**									
**1**	FCL: 10	0	FCL: 50	0	0	0	92,5	87.5	92.6	83.3
**2**	FCL: 20	0	0	0	0	0	98.4	96.6	98.1	98.3
**3**	FCL: 20	0	FCL: 20	0	0	0	100.0	96.6	99.4	99.8
**4**	FCL: 20	0.1	FCL: 20	0.1	0	0	100.0	97.3	99.4	99.8
**5**	FCL: 20	0.2	FCL: 20	0.2	0	0	100.0	96.6	99.2	99.8
**6**	FCL: 20	0.3	FCL: 20	0.3	0	0	100.0	96.6	99.1	99.8
**7**	FCL: 30	0	FCL: 0	0	0	0	99.3	96.6	99.0	99.2
**8**	FCL: 30	0	FCL: 30	0	0	0	100.0	96.6	99.2	99.8
**9**	FCL: 30	0.1	FCL: 30	0.1	0	0	100.0	97.3	99.4	99.9
**10**	FCL: 30	0.2	FCL: 30	0.2	0	0	100.0	98.0	99.6	99.9
**11**	FCL: 30	0.3	FCL: 30	0.3	0	0	99.3	98.0	99.2	99.9
**12**	FCL: 50	0	FCL: 50	0	0	0	95.5	93.4	96.3	96.3
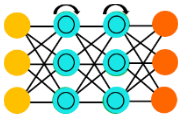	**LSTM (Long-Short Term Memory)**									
**13**	LSTMs: 20	0	FCL: 20	0	0	0	100.0	98.0	99.6	99.9
**14**	LSTMs: 20	0	LSTMs: 20	0	FCL: 20	0	100.0	98.0	99.6	99.9
**15**	LSTMs: 30	0	FCL: 20	0	0	0	100.0	98.0	99.6	99.9
**16**	LSTMs: 30	0	LSTMs: 30	0	FCL: 20	0	100.0	97.3	99.4	99.9
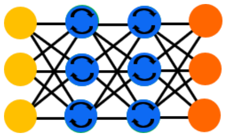	**BLSTM (Bidirectional Long-Short Term Memory)**									
**17**	B- LSTM:20	0	FCL: 20	0	0	0	100.0	97.3	99.4	99.9
**18**	B- LSTM:20	0	B- LSTM:20	0	FCL: 20	0	100.0	97.3	99.4	99.9
**19**	B- LSTM:30	0	0	0	0	0	100.0	97.3	99.4	99.9
**20**	B- LSTM:30	0	B- LSTM:30	0	FCL: 20	0	100.0	98.0	99.4	99.9
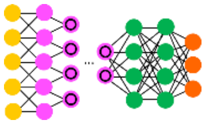	**CNN (Convolutional)**									
**21**	CNN:10	0	FCL:50	0	0	0	88.5	87.3	91.3	92.3
**22**	CNN:10	0	FCL: 30	0	0	0	91-4	93.6	93.4	95.6
**23**	CNN:20	0	FCL: 20	0	0	0	100.0	99.3	100.0	99.3
**24**	**CNN:20**	**0**	**CNN:20**	**0**	**FCL: 20**	**0**	**100.0**	**100.0**	**100.0**	**100.0**

Input Cell Output Cell Hidden Cell Memory Cell Convolution or Pool Bidirectional Cell

Note: Architectures were trained using Adam optimizer [8] with a batch size of 16 and 200 epochs. It was set an initial learning rate of 0.001.

Table S3. F_1 Score for each fold in MLP architecture.

**Fold**	1	2	3	4	5	6	7	8	9	10	**Mean**
**F1 Score**	1.00	1.00	0.91	0.89	0.83	0.91	0.91	0.80	1.00	0.91	**0.92**

Table S4 . F_1 Score for each fold in CNN architecture.

Fold	1	2	3	4	5	6	7	8	9	10	Mean
F1 Score	1.00	1.00	1.00	1.00	1.00	1.00	1.00	1.00	1.00	1.00	**1.00**

Table S5. F_1 Score for each fold in LSTM architecture.

Fold	1	2	3	4	5	6	7	8	9	10	Mean
F1 Score	1.00	1.00	1.00	0.89	0.91	1.00	0.89	1.00	1.00	1.00	**0.97**

Table S6. F_1 Score for each fold in B- LSTM architecture.

Fold	1	2	3	4	5	6	7	8	9	10	Mean
F1 Score	1.00	1.00	0.91	0.89	0.91	1.00	1.00	1.00	0.91	1.00	**0.96**

Table S7. Performance of the 1D-CNN architecture fed with different combinations of data groups. The most relevant data are marked in bold.

Group	Accuracy in training (%)	Accuracy in validation (%)	Sensitivity (%)	Specificity (%)
$G_{1,2}$	89.96	74.66	72.0	78.9
$G_{1,3}$	93.87	87.33	86.0	88.0
$G_{2,3}$	93.19	86.66	88.0	90.0
$G_{2,4}$	98.97	96.66	96.0	98.0
$G_{3,4}$	99.0	97.30	98.0	96.0
$G_{4,1}$	**99.8**	**99.3**	**98.00**	**97.0**

Table S8. Results of the training process using unseen data (employed model: 1D-CNN).

Accuracy					
**Training**	**Validation**	**Testing**	**Sensitivity**	**Specificity**	**F1 score**
1.00	1.00	1.00	1.00	1.00	1.00

Supporting References

(1) *Principal Component Analysis (PCA) Explained | Built In*. https://builtin.com/data-science/step-step-explanation-principal-component-analysis (accessed 2023-06-28).
(2) Verma, Y. *A Complete Understanding of Dense Layers in Neural Networks*. Analytics India Magazine. https://analyticsindiamag.com/a-complete-understanding-of-dense-layers-in-neural-networks/ (accessed 2023-07-06).
(3) Ajit, A.; Acharya, K.; Samanta, A. A Review of Convolutional Neural Networks. In *2020 International Conference on Emerging Trends in Information Technology and Engineering (ic-ETITE)*; 2020; pp 1–5. https://doi.org/10.1109/ic-ETITE47903.2020.049.
(4) *Long Short-Term Memory Network - an overview | ScienceDirect Topics*. https://www.sciencedirect.com/topics/computer-science/long-short-term-memory-network (accessed 2023-07-14).

(5) Ting, K. M. Confusion Matrix. In *Encyclopedia of Machine Learning*; Sammut, C., Webb, G. I., Eds.; Springer US: Boston, MA, 2010; pp 209–209. https://doi.org/10.1007/978-0-387-30164-8_157.
(6) *sklearn.metrics.confusion_matrix*. scikit-learn. https://scikit-learn/stable/modules/generated/sklearn.metrics.confusion_matrix.html (accessed 2023-10-05).
(7) *F1 Score in Machine Learning: Intro & Calculation*. https://www.v7labs.com/blog/f1-score-guide, https://www.v7labs.com/blog/f1-score-guide (accessed 2023-10-05).
(8) Kingma, D. P.; Ba, J. Adam: A Method for Stochastic Optimization. arXiv January 29, 2017. https://doi.org/10.48550/arXiv.1412.6980.
(9) Redelinghuys, M. J.; Geldenhuys, J.; Jung, H.; Kock, M. M. Bacterial Vaginosis: Current Diagnostic Avenues and Future Opportunities. *Front. Cell. Infect. Microbiol.* **2020**, *10*. https://doi.org/10.3389/fcimb.2020.00354.
